# Development of User-Friendly Method to Distinguish Subspecies of the Korean Medicinal Herb *Perilla frutescens* Using Multiplex-PCR

**DOI:** 10.3390/molecules22040665

**Published:** 2017-04-21

**Authors:** Yonguk Kim, Ah-Young Kim, Ara Jo, Hakjoon Choi, Seung-Sik Cho, Chulyung Choi

**Affiliations:** 1 Jeonnam Institute of Natural Resources Research, Jangheung-gun, Jeollanamdo 59338, Korea; kyu9801@hanmail.net (Y.K.); joara9153@naver.com (A.J.); ohchj12@naver.com (H.C.); 2 School of Biological Sciences and Technology, Chonnam National University, Gwangju 61186, Korea; kimay1243@hanmail.net; 3 Department of Pharmacy, College of Pharmacy, Mokpo National University, Muan, Jeonnam 58554, Korea; sjason1@naver.com

**Keywords:** simple sequence repeat (SSR), Myb-P1, dihydroflavonol 4-reductase (DFR), Perilla, Jasoyeop, indel marker

## Abstract

Perilla (*Perilla frutescens*) is an economically and culturally important plant in East Asia. Plant breeding between cultivars has enhanced the genetic diversity of perilla overall, but means that functionally diverse subspecies are more difficult to identify and distinguish. In this study, we developed gene-based DNA markers to distinguish between the Korean herbal medicinal perilla varieties. We identified informative simple sequence repeat (SSR) regions on the promoter regions of the *Myb*-*P1* and dihydroflavonol 4-reductase (*DFR*) genes, as well as a large insertion-deletion (indel) region in the limonene synthase (*LS*) gene, and developed markers to characterize the distinct subspecies differences (*PfMyb*-*P1pro*, *PfDFRpro*, and *PfLS*, respectively). Using the *PfLS* primers, a 430-bp region could be amplified from *P*. *frutescens* var. *acuta*, *crispa*, and f. *viridis* (known as Jasoyeop, Jureum-soyeop, and Chungsoyeop, respectively), but not from *P*. *frutescens* var. *japonica* (Dlggae). The *PfMybpro* primers resulted in PCR products of 314 or 316, 330, 322, and 315 bp from Dlggae, Jasoyeop, Jureum-soyeop, and Chungsoyeop, respectively, and the *PfDFRpro* primers resulted in products of 189 or 202, 187 or 189, 185 or 189, and 193bp, respectively, for the four perilla subspecies. Combining these three reactions into a single multiplex PCR approach resulted in subspecies-specific PCR band patterns for six common types of commercial perilla, distinguishing between three varieties of Dlggae (Cham-Dlggae, Ip-Dlggae, and Bora-Dlggae), as well as identifying Jasoyeop, Jureum-soyeop, and Chungsoyeop. These user-friendly markers will be valuable as a simple and efficient method for identifying the Korean medicinal herb Jasoyeop, as well as distinguishing between other functionally distinct subspecies, which may have broad applications in the Korean herbal industry.

## 1. Introduction

Perilla (*Perilla frutescens*) is an economically, culturally, and medicinally important annual in the Labiatae family, native to East Asia. It is cultivated primarily in China, India, Japan, Korea, Thailand, Turkey, and Vietnam, where it is prized for its multiple uses, including as an oilseed (*P. frutescens* var. *japonica* (Hassk.) H. Hara, known as ‘Ren’ in Chinese, ‘Dlggae’ in Korean, and ‘Egoma’ in Japanese), a leaf vegetable crop (‘Kkaennip’ in Korean), and a medicinal herb (‘Zisu’ in Chinese, ‘Cha-jo-ki’ or ‘so-yeop’ in Korean, and ‘Jiso’ in Japanese) [1,2,3]. Its recognized bioactivities include antioxidant [4,5], anti-allergic [6,7,8], anti-inflammatory [6,7,8,9], antipruritic [9], and anti-HIV-1 [10] activity. It is also a common ingredient in foods such as pickles (‘Shiso’ in Japanese), with two different leaf colors caused by their differing accumulation of anthocyanins [11,12,13]: a red-purple type (red perilla, *P*. *frutescens* var. *acuta* (Odash.) Kudo and *crispa* (Benth.) W. Deane, Jasoyeop in Korean, Aka-jiso in Japanese) and a green-purple type (green perilla, *P*. *frutescens* f. *viridis* Makino, ‘Chungsoyeop’ in Korean, ‘Ao-jiso’ in Japanese).

Perilla varieties are commonly classified into seven chemo-types based on their varying quantities of the main components of its essential oil, perillaldehyde (PA), perilla ketone (PK), elsholtzia ketone (EK), citral (C), perillene (PL), piperitenone (PT), and phenylpropanoid (PP) [14,15]. In Korea, the Perilla genus has been classified into two major chemo-types, PA (soyeop) and PK (Dlggae), according to the main components of their volatile oils. PA plants contain mostly perillaldehyde (19.8%) and limonene (57.7%), while the oil of PK plants is mainly perilla ketone (89.8%) [16]. The leaves and seeds of PK plants have traditionally been used as food, whereas the leaves and twigs of PA plants are used in herbal medicine. 

Recently, Korean periila breeders have developed new and improved perilla varieties with high yield and nutritional quality by crossing between subspecies. These diverse varieties of PA and PK perilla are difficult to distinguish morphologically, and the accurate and rapid identification of these varieties using the nuclear ribosomal internal transcribed spacer (ITS) region or chloroplast DNA regions (matK, psbA-trnH, and trnL-trnF) is limited by the relatedness of the subspecies (Table S1). If they should be used in herbal medicine and as a health food supplement, the development of accurate methods for the identification and characterization of these varieties at the subspecies level is required. Previous studies have reported genetic differences between cultivated and weedy forms of perilla using random amplified polymorphic DNA and amplified fragment length polymorphism markers; however, the accuracy of these analyses is limited by population dynamics and their reproducibility as the diagnostic markers of perilla in the field [1,2].

Recent advances in next-generation sequencing and RNA sequencing have provided not only a fast, cost-effective, and reliable approach to generate large datasets for functional genomic and transcriptomic analysis, but also more comprehensive and efficient means to quantify specific expression patterns between subspecies. These new techniques have been applied to red and green perilla varieties, yielding useful information for transcript discovery, and expression profiling, highlighting differences between the two genotypes [17,18,19]. In the present study, we referred to data for the transcriptomic differences identified between the red and green PA perilla varieties [17,18] to select putative informative genes and establish simple sequence repeat (SSR) and insertion-deletion (indel) markers to enable breeders to readily distinguish between perilla varieties. SSR markers have been widely used as genetic markers, which are very useful for a spectrum of genetic and breeding applications because of their abundance, reproducibility, high level of polymorphism, co-dominance inheritance, and easy detection by PCR [20,21]. 

We selected three candidate genes, *LS*, *Myb*-*P1*, and *DFR*, among the identified differentially expressed genes for perilla plants that feature red and green forms of anthocyanin accumulation from RNA-seq data [17,18]. This genes are, respectively, involved in the biosynthesis of limonene from the universal acyclic precursor geranyl diphosphate (GPP), a major enzyme in the biosynthesis of perillaldehyde produced mainly in PA types, and two regulators of anthocyanin biosynthesis and drought stress acclimation of red-colored perilla [22,23]. We successfully revealed PA-type subspecies-specific markers using three of the candidate genes, *LS*, *Myb*-*P1*, and *DFR*, from 12 perilla cultivars in Korea (Table 1) and developed them for use in an optimized multiplex PCR. This approach enabled the detection and identification of PA-type varieties of *P*. *frutescens* in Korean Jasoyeop herbal products, resulting in a user-friendly, efficient, and accurate method to distinguish between perilla subspecies.

## 2. Results and Discussion

To identify subspecies-specific genomic regions in the PA and PK perilla varieties, the genome and promoter sequences of three previously identified [17,18] candidate genes, *LS*, *Myb*-*P1*, and *DFR*, were downloaded from NCBI. Based on PCR profiling derived from these sequences using 44 primer combinations (Table 2), three genes (*LS*, *Myb*-*P1*, and *DFR*) were found to contain distinct indel and SSR patterns that varied between the perilla varieties.

The primer pairs were developed from nucleotide or promoter sequences containing coding, encoding, 5′ and 3′ UTR regions. These sequences were identified from source sequence Genbank among PA type of perilla.

**Figure 1:**
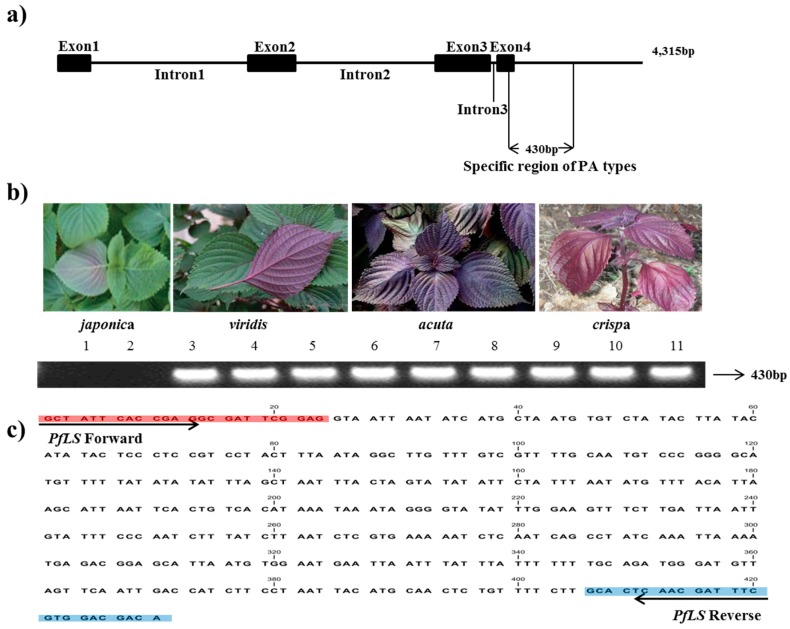
Development of the *PfLS* marker. (**a**) *LS* region specific to PA-type genomes identified in *P*. *frutescens* var. *crispa*, *acuta*, and *viridis*. The amplified region covered part of exon 4 and 3′ UTR; (**b**) Application of the *PfLS* marker in the commercial breeding perilla lines (1–2: *P*. *frutescens* var. *japonica*; 3–5:f. *viridis*; 6–8: var. *acuta*; 9–11: var. *crispa*). (**c**) 430 bp sites of specific target region.

In the 4,315-bp *LS* sequence of the PA genotype, the four primer pairs (LS1-4) covered an approximately 0.9–1.0-kb region conserved between the PA and PK types (Table 2). Two primer pairs (LS6 and LS7 F/R2) amplified a region with a subspecies-specific difference in sequence length, amplifying 1012 bp in PA and 582 bp in PK between exon 3 and 3′ UTR. Indel DNA fragments were identified by sequencing and aligning the DNA sequences of this region from two accessions of *P*. *frutescens* var. *japonica*, as well as three accessions each of *P*. *frutescens* var. *acuta*, *crispa*, and *P*. *frutescens* f. *viridis*, with the published *LS* sequence (GenBank accession number: AF241791). This event was identified as a deletion of 430 bp in var. japonica across exon 4 and 3′ UTR; therefore, we considered the 430-bp indel site to be a unique identifier of the PA type. The *PfLS* primer pair generated a single, clear band in the PA-type accessions (Figure 1, Table 3).

Perillaldehyde and limonene has been reported as major volatile compounds and marker components in *P*. *frutescens* var. *acuta* grown in East asia. Ohk et al. [16] were reported that the contents of major component in PA chemotype grown in Korea analyzed 57.9% of limonene and 19.6% of perillaldehyde. Based on this result, we obtained the PA type specific indel region from limonene synthase (LS) gene by marker-based approaches. Therefore, we proved that limonene is a chemical major component as well as a subspecies specific marker between PA and PK types at the genetic level. 

**Figure 2:**
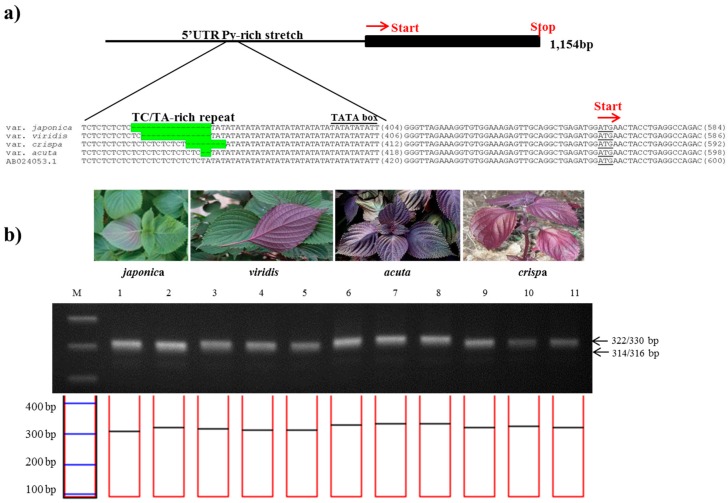
Development of the *PfMyb*-*P1pro* marker. (**a**) Identification of a simple sequence repeat (SSR) in the promoter region of the *Myb*-*P1* gene. The sequence alignment shows the SSR variation of the 5′-UTR Py-rich stretch and AT-rich regions in the promoter of *Myb*-*P1* among four subspecies of *P*. *frutescens*. The SSR variations are highlighted in green; (**b**) Application of the *PfMyb*-*P1pro* marker in the commercial breeding perilla lines. (M: 100bp DNA ladder; 1–2: *P*. *frutescens* var. *japonica*; 3–5: f. *viridis*; 6–8: var. *acuta* Kudo; 9–11: var. *crispa*).

The 1,141-bp sequence of the *Myb*-*P1* gene was sequenced from two accessions of *P*. *frutescens* var. *japonica* and nine accessions each of *P*. *frutescens* var. *acuta*, *crispa*, and f. *viridis*, then aligned with the previously published sequence (GenBank accession number: AB024053). A 657 bp region of the 5′ upstream promoter of *Myb*-*P1* (Myb-P1pro2 primers) was found to have an informative nuclear SSR in a TA-rich region contained within a 5′-untranslated region (UTR) Py-rich sequence, a cis-acting element conferring high transcription levels (Table 2). To confirm the best amplification patterns for this polymorphic region, the observed product size including the number of alleles amplified and PIC value for *Myb-P1pro* marker used in this study is provided in Table 3. *Myb-P1pro* marker produced five alleles for all perilla accessions. As shown in Figure 2, the amplicon sizes of *Myb*-*P1pro* marker were 314 or 316 bp from *P*. *frutescens* var. *japonica*, whereas amplicons from *P*. *frutescens* var. *acuta*, *crispa*, and f. *viridis* were 330, 322, and 315 bp respectively (Table 2). The amplicon sizes showed clear differences among the three subspecies, while there was no difference between *P*. *frutescens* var. *japonica* and f. *viridis*.

Gong et al. [24] suggested that *Myb-P1* is a key factor in the regulation of anthocyanin biosynthesis and the main factor responsible for determining anthocyanin formation in red P. frutescens. Fukushima et al. [17] reported that *Myb-P1* is a highly upregulated gene associated with the anthocyanin biosynthesis and accumulation from red forms of perilla plants by RNA seq data. Therefore, according to these results, we performed the subspecies-specific SSR region from *Myb-P1* promoter region, whereas there was no significant difference in coding and encoding region of *Myb-P1* gene sequence.

**Figure 3:**
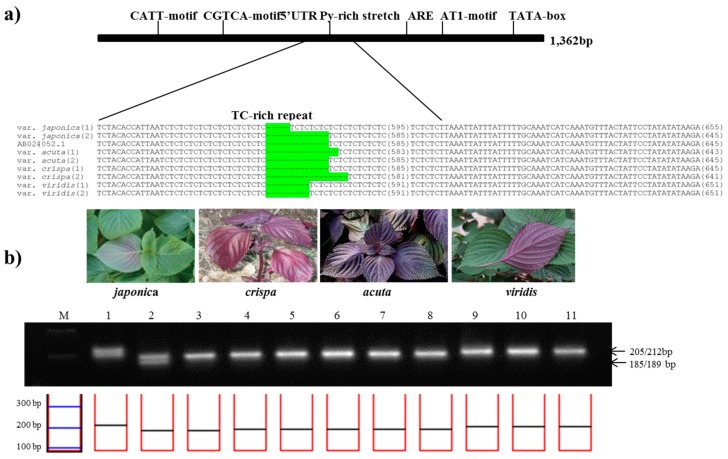
Development of the *PfDFRpro* marker. (**a**) Identification of a simple sequence repeat (SSR) in the promoter region of the *DFR* gene. The sequence alignment shows the SSR variation of 5′-UTR Py-rich stretch in the promoter of *DFR* among four subspecies of *P*. *frutescens*. The SSR variations are highlighted in green; (**b**) Application of the *PfDFRpro* marker in the commercial breeding perilla lines. (M: 100bp DNA ladder; 1–2: *P*. *frutescens* var. *japonica*; 3–5: var. *crispa*; 6–8: var. *acuta*; 9–11:f. *viridis*).

In the same manner as described above, among total 1,364 bp of DFR sequence, a 745 bp region of the subspecies-specific nuclear SSR (DFRpro1 primers) was identified in aligned region with 5′-UTR Py-rich stretch between 11 perilla accessions (Table 2). To confirm the best amplification patterns for this polymorphic region, the observed product size including the number of alleles amplified and PIC value for *DFRpro* marker used in this study is provided in Table 3. *DFRpro* marker produced seven alleles for all perilla accessions. As shown in Figure 3, the product sizes for the *DFRpro* primers were as follows: 189 or 202 bp from *P*. *frutescens* var. *japonica*, 187 or 189 bp in *acuta*, 185 or 189 bp in *crispa*, and 193 bp in f. *viridis*. Although the amplicon sizes were similar between *P*. *frutescens* var. *japonica*, *acuta*, and *crispa*, the *DFRpro* marker could clearly distinguish these from Chungsoyeop, *P*. *frutescens* f. *viridis* (Table 3). 

Dihydroflavonol reductase (*DFR*) gene known as one of the vital factor for the formation of anthocyanin, since the mutants lacking this enzyme do not produce anthocyanin [24,25]. Interaction between *Myb-P1* and the promoter of gene encoding *DFR* from P. frutescens was shown that the expression of Myb-P1 was 10-fold abundant in red perilla than in green perilla as well as remarkably induced by light [25]. These data suggest that *DFR* gene may also be involved in formation and regulation of anthocyanin biosynthesis as well as the significant factor for determination of anthocyanin form in red and green perilla.

Based on above results, although we confirmed the subspecies-specific SSR marker for *P*. *frutescens* f. *viridis* from *DFR* promoter region, there was no significant difference between perilla subspecies.

**Figure 4:**
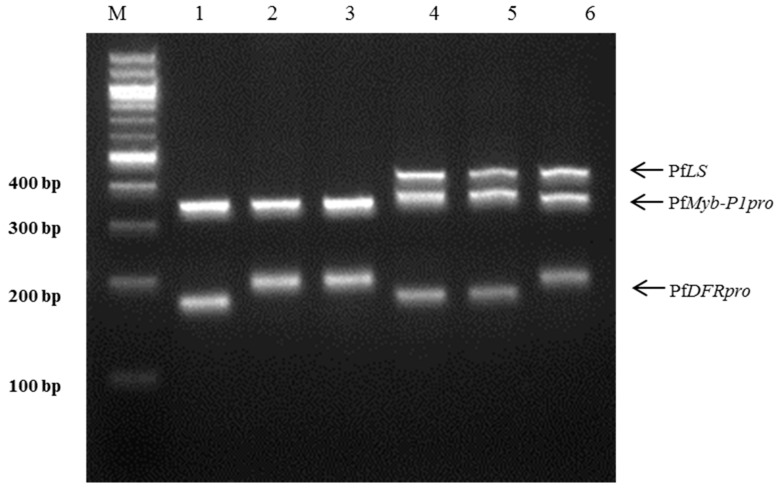
Multiplex PCR assay using three specific markers for the perilla subspecies in a single reaction. A mixture of three specific markers, *PfLS*, *PfMyb*-*P1pro*, and *PfDFRpro*, was used for PCR amplification. Lanes on 3% electrophoresis gel: M: 100 bp DNA ladder; 1–3: ‘Cham-Dlggae’, ‘Ip-Dlggae’, and ‘Bora-Dlggae’, respectively, which represent the three cultivar types of *P*. *frutescens* var. *japonica*; 4: ‘Jureum-soyeop’, representing *P*. *frutescens* var. *crispa*; 5: ‘Jasoyeop’, representing *P*. *frutescens* var. *acuta*; 6: ‘Chungsoyeop’, representing *P*. *frutescens* f. *viridis*.

The *PfLS*, *PfMyb*-*P1pro*, and *PfDFRpro* primers were used to develop a multiplex-PCR assay to distinguish the Korean herbal medicinal plant ‘Jasoyeop’ (*P*. *frutescens* var. *acuta*). The primer specificity, concentration, and melting temperature (Tm) are very crucial in the development of multiplex PCR since its success depends on the capacity of the primers to be relatively annealed with their targets under a single set of PCR conditions [26,27]. Optimal amplication conditions were determined by altering the following parameters: annealing time (85–90 s), annealing temperature (58–60 °C), primer concentration (0.5, 1.0, and 1.0 μM of each primer). Amplification with this combination of primers yielded distinct DNA fragments with two or three multi-bands that corresponded to the amplicons produced by the individual indel and SSR reactions. The amplicon sizes were used to identify the subspecies. Multiplex PCR with the three primer pairs clearly discriminated the six commercial perilla cultivars: three varieties of *P*. *frutescens* var. *japonica* (Cham-Dlggae, Ip-Dlggae, and Bora-Dlggae), and one variety each of *P*. *frutescens* var. *crispa* (Jureum-soyeop), *acuta* (Jasoyeop), and f. *viridis* (Chungsoyeop). The PCR products of the three *P*. *frutescens* var. *japonica* varieties were specific double-band amplicons: Cham-Dlggae had bands at 189 and 316 bp, Ip-Dlggae at 202 and 314 bp, and Bora-Dlggae at 202 and 316 bp, corresponding to *PfDFRpro* and *PfMyb*-*P1pro*, respectively. The PCR amplified three distinct products from the other three subspecies: *P*. *frutescens* var. *crispa* showed bands at 189, 322, and 430 bp; *P*. *frutescens* var. *acuta* showed bands at 189, 330, and 430 bp, and *P*. *frutescens* f. *viridis* had bands at 193, 315, and 430 bp. The bands corresponded to *PfDFRpro*, *PfMyb*-*P1pro*, and *PfLS*, respectively (Figure 4, Table 2).

**Figure 5:**
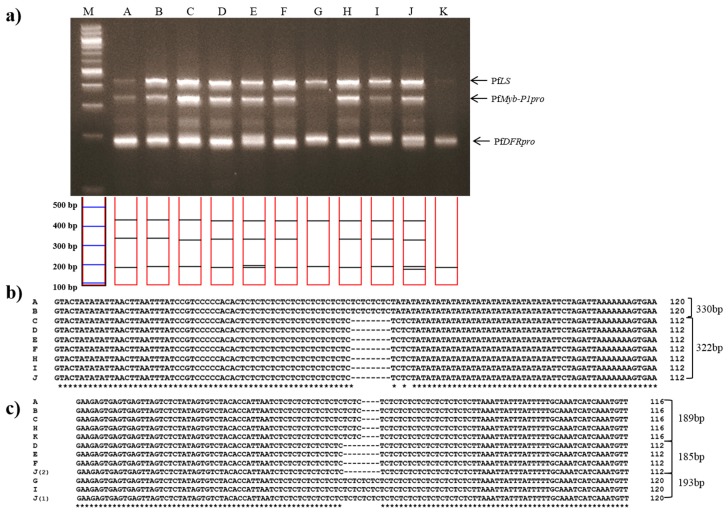
Multiplex PCR identification of 11 commercial dried leaves and twigs of Jasoyeop products. (**a**) Lanes on 3% electrophoresis gel: M: 100 bp DNA ladder; A–K: purchased commercial dried Jasoyeop products (see Table 3 for full details); (**b**,**c**) Sequence analysis of PCR products amplified using the *PfMybpro* and *PfDFRpro* marker primers, respectively, aligned using Clustal W2.

Jasoyeop, a mixture of dried leaves and twigs of either *P. frutescens* var. *acuta* or *crispa*, is regularly consumed as a tea and herbal supplement in Korea. To validate the utility of multiplex PCR in commercial dried herbal materials, genomic DNA was extracted from leaf samples of 11 Jasoyeop products and amplified using the subspecies-specific primers. Almost all of the samples gave rise to clear triple band patterns consistent with the *P*. *frutescens* var. *acuta* or *crispa*, which are commonly used in *Jasoyeop* products (Figure 5a). The amplified PCR products were sequenced to confirm the identification of *P*. *frutescens* var. *acuta* (Figure 5b,c). As shown in Figure 5a, the band patterns and sequences revealed that A and B are *P*. *frutescens* var. *acuta*, while C-F, H, and J were identified as being *P*. *frutescens* var. *crispa*. Although G and I showed similar banding patterns to the other samples, they were determined not to be *crispa* nor *acuta* by their mixed size ranges; their *PfDFRpro* amplicons resembled *P*. *frutescens* f. *viridis* while their *PfMyb*-*P1pro* amplicon resembled *P*. *frutescens* var. *crispa*. Sample K was also not identified because only the 189 bp amplicon of *PfDFRpro* was detected. The sensitivity of the multiplex system was checked using template DNA dilution methods [27]. Optimized template DNA concentration isolated from fresh leaves of perilla was 10–20 ng. Therefore, the gel image (Figure 4a) clearly detected all perilla subspecies. However, Figure 5a shows that the three multi bands for lane G and K was difficult to detect when 10 ng DNA templates isolated from dried leaves of herbal products were used. Although these bands remained undetected in this study, our results demonstrated the specificity and sensitivity of these subspecies-specific primers, which were used to identify and distinguish between PK- and PA-type perilla varieties.

The Korean Food and Drug Administration noted that the two perilla medicinal varieties, *P*. *frutescens* var. *acuta* and *crispa* were established as Jasoyeop and are not currently distinguishable in herbal medicine [28]. Nevertheless, for the standardization efforts to control the quality of the medicinal plant, the distinction between *P. frutescens* var. *acuta* and *crispa* may be required. Nuclear ITS and chloroplast DNA sequences have commonly been used to develop molecular markers to identify various plant species [29]; however, the limited differences between perilla subspecies at these loci do not provide sufficient information to distinguish between subspecies (Table S1).

Using previously identified genes putatively providing distinct genetic differences between perilla cultivars [17,18], we identified subspecies-specific indels and SSRs in three genes and developed *PfLS*, *PfMyb*-*P1pro*, and *PfDFRpro* markers to distinguish between the four Korean perilla varieties, Dlggae (*P*. *frutescens* var. *japonica*), Chungsoyeop (*P*. *frutescens* f. *viridis*), Jasoyeop (*P*. *frutescens* var. *acuta*), and Jureum-soyeop (*P*. *frutescens* var. *crispa*), using indel and SSR sites identified from the exon4/3′ UTR regions of *LS* and the 5′ upstream promoter regions of *Myb*-*P1* and *DFR*.

## 3. Materials and Methods

### 3.1. Plant Materials and Growth Conditions

A total of 80 samples from 12 perilla accessions (three of *P*. *frutescens* var. *japonica*, *acuta*, *crispa*, and *P*. *frutescens* f. *viridis*, respectively) were analyzed in this study (Table 1). Seeds were purchased from different commercial seed companies or collected from their native habitats in Korea and germinated at 25 °C under illumination in a glasshouse under a 16-h light period. After about 7–8 weeks, DNA was extracted from the young leaves. Herbal medicines were purchased from herbal markets in different geographical regions. Dried leaves and twigs of Jasoyeop products (mixed *P*. *frutescens* var. *acuta* or *crispa*) were purchased at local markets (Table 4) and used for the extraction of DNA.

### 3.2. Preparation of Genomic DNA

Total genomic DNA was extracted from 0.2 to 1.0 mg fresh and dried leaves using a Plant DNeasy extraction kit (Qiagen, Hilden, Germany), following the manufacturer′s protocol. The quantity and quality of the extracted DNA were determined using a Nanodrop ND-1000 (Thermo Fisher Scientific, Wilmington, DE, USA), after which it was diluted to approximately 10–20 ng·μL^−1^ for PCR and multiplex-PCR amplification and stored at −20 °C until required.

### 3.3. Gene Profiles and PCR Product Sequencing

In a previous study, Yuba et al. [14] reported limonene synthase (LS) full genome sequence from *P*. *frutescens*. Based on Gong et al. [12], we applied full genome of Myb-P1 and DFR promoters shown distinct differences between Perilla red- and green-colored types. To identify genomic regions containing subspecies-specific sequences for the development of markers based on indel mutations or SSRs, gene profiling was performed using gene-specific primers (Table 3). A total of 19 25-mer primer combinations were designed using the nucleotide and promoter sequences of LS, Myb-P1, and DFR, taken from NCBI GenBank (accession nos.: AF241791, AB024053, and AB024052). These primers were used to perform a PCR of the extracted genomic DNA using Ex Taq DNA polymerase (Takara, Kyoto, Japan). The PCR mixture consisted of 10–20 ng genomic DNA, 4nmol each dNTP, 10pmol each primer, 1 unit Ex. Taq DNA polymerase and 2 μL Ex. Taq buffer (TaKaRa, Kyoto, Japan) in a total volume of 20 μL. The PCR amplification was carried out under the following conditions: an initial denaturation at 95 °C for 5 min; 40 cycles of denaturation at 95 °C for 30 s, annealing at 58 °C for 30 s, and extension at 72 °C for 2 min; ending with a final extension at 72 °C for 5 min. The resulting products were separated by 2% agarose gel electrophoresis, and the target DNA was extracted and purified using a QIAquick Gel Extraction kit (Qiagen, Hilden, Germany). The purified PCR products were introduced into a TA cloning vector using the Topcloner™ TA kit (Enzynomics, Daejeon, Korea), and the ligation products were transformed into Escherichia coli DH5-α competent cells. The recombinant plasmids were purified using a Plasmid Mini kit (Qiagen, Hilden, Germany), and sequenced at Cosmo Genetech (Daejeon, Korea). Three colonies were sequenced for each variety, providing three biological repeats. 

### 3.4. Development of Specific Markers and Data Analysis

The 16 *LS*, 12 *Myb*-*P1*, and 6 *DFR*, gene sequences obtained from the eight varieties were assembled, edited, and aligned using MEGA 6.0 and Clustal W2 [30,31]. The contigs were aligned to determine any regions containing subspecies-specific indels or SSRs. PCR reactions were performed to confirm primer specificity, using the following conditions: an initial denaturation at 94 °C for 5 min; followed by 40 cycles of denaturation at 94 °C for 30 s, annealing at 62 °C for 30 s, and extension at 72 °C for 30 s; with a final extension for 5 min at 72 °C. The amplified fragments were checked using a 3.0% MetaPhor agarose gel (Lonza, Rockland, ME, USA). The size of each amplification product was automatically estimated using the UVIsoft image analyzer system. For the band size measurements, a charge-coupled device camera imaging system and UVIsoft analysis (Gel documentation and analysis systems, Uvitec, Cambridge, UK) were used to capture the image and to calculate the band size. The polymorphic information content (PIC) was calculated for each fragment using the formula PIC*ὶ* = 2ƒ*ί*(1 − ƒ*ὶ*), where ƒ*ὶ* is the frequency of amplified fragments (bands that are present), and (1 − ƒ*ὶ*) is the frequency of non-amplified fragments (bands that are absent) of marker *ὶ*. The PIC value is a measure of how many alleles a marker generates on locus [32]. In this study, two SSR markers is considered as an allele for the purposes of calculations.

### 3.5. Development of the Multiplex-PCR Assay

Following the Multiplex PCR kit protocol (Qiagen, Hilden, Germany), three subspecies-specific primer sets derived from the *LS*, *Myb*-*P1*, and *DFR* genes were combined into a single PCR reaction. Optimal PCR conditions were determined by altering the annealing temperature (58–62 °C), the concentration of each primer (0.5–2.0 μM), and using various combinations of primers. Genomic DNA samples isolated from commercial perilla cultivars and dried Jasoyeop products were amplified in a multiplex PCR mix containing 25 μL 2x Qiagen Multiplex PCR master mix, 5 μL 5x Q-solution, 20 ng template DNA, and 5 μL 10x primer mix (final primer concentration: 0.5 μM *PfLS*, 1 μM *PfMyb*-*P1*, and 1 μM *PfDFR*). The PCR conditions were as follows: an initial denaturation at 95 °C for 5 min; followed by 40 cycles of denaturation at 95 °C for 30 s, annealing at 60 °C for 90 s, and extension at 72 °C for 90 s; with a final extension for 10 min at 68 °C.

## 4. Conclusions

We successfully developed a multiplex PCR assay combining the three markers, which amplifies three distinct DNA fragments to distinguish between these four subspecies in a single reaction. These marker combinations can be used to efficiently distinguish between the PA- and PK-types of perilla in Korea and to discriminate the medicinal herbal plant Jasoyeop (*P*. *frutescens* var. *acuta*) using fresh or dried plant material, and will therefore enable the analysis of Jasoyeop products in the herbal market.

## Figures and Tables

**Table 1:** Plant materials used in this study.

No.	Scientific Name	Common Name	Collection	Location
**1**	*P. frutescens* var. *japonica*	Cham-Dlggae	Yongin-si, Gyeonggi-do, Korea	37°06′32.7″N 127°07′09.7″E
**2**		Ip-Dlggae	Yongin-si, Gyeonggi-do, Korea	37°06′32.7″N 127°07′09.7″E
**3**		Bora-Dlggae	Hwasun-gun, Jeollanam-do, Korea	35°02′47.5″N 126°57′13.9″E
**4**	*P. frutescens* var. *crispa*	Jureum-soyeop	Hwasun-gun, Jeollanam-do, Korea	35°02′47.5″N 126°57′13.9″E
**5**			Jangheung-gun, Jeollanam-do, Korea	34°40′03.0″N 126°56′44.6″E
**6**			Yongin-si, Gyeonggi-do, Korea	37°06′32.7″N 127°07′09.7″E
**7**	*P. frutescens* var. *acuta*	Jasoyeop	Yecheon-gun, Gyeongsangbuk-do, Korea	35°59′28.1″N 128°55′33.3″E
**8**			Namyangju-si, Gyeonggi-do, Korea	37°38′58.1″N 127°11′31.7″E
**9**			Hwasun-gun, Jeollanam-do, Korea	35°02′47.5″N 126°57′13.9″E
**10**	*P. frutescens* f. *viridis*	Chungsoyeop	Yecheon-gun, Gyeongsangbuk-do, Korea	35°59′28.1″N 128°55′33.3″E
**11**			Namyangju-si, Gyeonggi-do, Korea	37°38′58.1″N 127°11′31.7″E
**12**			Hwasun-gun, Jeollanam-do, Korea	35°02′47.5″N 126°57′13.9″E

**Table 2:** Primer pairs tested in PCR with genomic DNA from 12 perilla cultivars.

Gene Name	Genbank Accession	Primer Sequences 5′ to 3′	Tm °C	Size (bp)	Location
**LS1 ^a^**	AF241791	F:GAATGTGCATTTGGAATATTATAAG R:TTGTGAAGCGTACGTTCTGCCGGAG	58	255	5′UTR
**LS2 ^a^**		F:CATCAGACGGTCTAACGGGTAACAA R1:CACTATGCTCTATAATTAATTGGGTG R2: AAATATATACCATAAAATGGATTAG R3:GTTTTGTCCGACACTAACGTGTGAG	60 59 61 58	253 515 576	Exon1 to Intron1
**LS3 ^a^**		F: GAATAGTTAATTAATGGGTGTGCAG R:AGGAGTCTGAATGCAAGAGCTGTCG	57	308	Exon2
**LS4 ^a^**		F: CCTGGTAGATACATATTTATATTAT R:TTTAGCACAGTGCACGAGCCACACG	60	189	Intron2
**LS5 ^a^**		F:CAACACTTGAACTGTGGCGGGTTGA R:ATGTCCAAAGAGTGGCGCACACATG	58	358	Exon3
**LS6 ^a^**		F: CTGGTGGATTGATGCCTATAAGAGG R: TACATACATACATATATAACATGAG	59	223	Exon3 to Intron4
**LS7 ^a^**		F: GCTATTCACCGAGGCGATTCGGAGG R1: ATTTTAATTTGATAGGCTGATTGAG R2:TGTCGTCCACGAAATCGTTGAGTGC	60 60	301 430	Exon4 to 3′UTR
**LS8 ^a^**		F: AGCGGCCCTGTAATGCTTTGCCATG R1:CACATCGCCTCGACTCACCTCCTCC R2:ATAAGCCATCTGATGTGGCGTCGCG	62 59	284 492	3′UTR
**Myb-P1pro1 ^b^**	AB024053	F: GAATTGGAAACAAATTAAGGATCGG R: TGATGTCTGGCCTCAGGTAGTTCAT	59	612	5′UTR
**Myb-P1pro2 ^b^**		F: TTAAGGATCGGAGATCGAATGAGG R: AGCTTGATCATCAGCTCTTCTTCAT	60	657	5′UTR
**Myb-P1pro3 ^b^**		F: ATGAACTACCTGAGGCCAGACATCA R: ATGAGTATGAATATTTCTTTGAGGT	58	601	Coding
**DFRpro1 ^c^**	AB024052	F: GAATTCGAGCTCGAATATGTACATT R: CGACCAACTATTATAGGCATAGCTCC	59	745	5′UTR
**DFRpro2 ^c^**		F: GGAGCTATGCCTATAATAGTTGGTCG R: GAAACAATCTTGACCACCATTCTTG	60	619	5′UTR

^a^ Limonene Synthase; ^b^ Myb-P1 promoter; ^c^ DFR promoter.

**Table 3:** Summary of subspecies-specific indel and two SSR markers used in the analysis of the 11 perilla accessions.

Marker Name	Marker Sequence		Product Size (bp)				Number of Alleles	PIC
			J ^a^	A ^b^	C ^c^	V ^d^		
**Pf*LS***	F	GCTATTCACCGAGGCGATTCGGAGG	N/A	430	430	430	N/A	N/A
	R	TGTCGTCCACGAAATCGTTGAGTGC						
**Pf*Mybpro***	F	GTACTATATATTAACTTAATTTATCCGT	314 316	330	322	315	5	0.23
	R	GCTTGATCATCAGCTCTTCTTCATCCAC						
**Pf*DFRpro***	F	GAAGAGTGAGTGAGTTAGTCTCTATAGT	189 202	187 189	185 189	193	7	0.36
	R	GCAAGAGATTTATGAGCATGTTCTAGAT						

^a^ *P. frutescens* var. *japonica*; ^b^ *P. frutescens* var. *acuta*; ^c^ *P. frutescens* var. *crispa*; ^d^ *P. frutescens* f. *viridis*.

**Table 4:** Herbal medicine material from 11 commercial dried Jasoyeop products used in this study.

Herbal Makers	Material Component	Source
**A**	Dried leaves and twigs of Jasoyeop or Jureum-soyeop products	Gapyeong-gun, Gyeonggi-do, Korea
**B**		Andong-si, Gyeongsangbuk-do, Korea
**C**		Yeongcheon-si, Gyeongsangbuk-do, Korea
**D**		Jecheon-si, Chungcheongbuk-do, Korea
**E**		Gapyeong-gun, Gyeonggi-do, Korea
**F**		Sancheong-gun, Gyeongsangnam-do, Korea
**G**		Taean-gun, Chungcheongnam-do, Korea
**H**		Yeongcheon-si, Gyeongsangbuk-do, Korea
**I**		Uiseong-gun, Gyeongsangbuk-do, Korea
**J**		Hunan Province, China
**K**		Yeongcheon-si, Gyeongsangbuk-do, Korea

## Supplementary Materials

The following are available. Table S1: Multiple sequence alignments of nucleotide and chloroplast DNA sequences in Perilla subspecies [33].
