# Supplementary material for: Development of User-Friendly Method to Distinguish Subspecies of the Korean Medicinal Herb Perilla frutescens Using Multiplex-PCR
Source: Molecules. 2017 Apr 21;22(4):665. doi: 10.3390/molecules22040665 (PMC6154563; doi:10.3390/molecules22040665)
Supplement: Supplementary file 1 [file molecules-22-00665-s001.pdf]

Supplementary Table 1. Multiple sequence alignments of nucleotide and chloroplast DNA sequences in Perilla subspecies.

Alignment was performed for a) ITS region (600bp), b) *matK* (951bp), c) *rbcL* (743bp), d) *trnH-psbA* (466bp), e) *rpoCF* (492bp), and f) ycf (381bp) using multiple sequence comparison by log expectation(MUSCLE v.3.4.31)[28].

a)

```
var. japonica     CGCGAACACGTGTTTAACATCATCGGACACGGCGTGGGGGAGACTCCCGTCGTGCACCGC   60
var. acuta        CGCGAACACGTGTTTAACATCATCGGACACGGCGTGGGGGAGACTCCCGTCGTGCACCGC   60
var. crispa       CGCGAACACGTGTTTAACATCATCGGACACGGCGTGGGGGAGACTCCCGTCGTGCACCGC   60
var. viridis      CGCGAACACGTGTTTAACATCATCGGACACGGCGTGGGGGAGACTCCCGTCGTGCACCGC   60
                  ************************************************************

var. japonica     TCCCGCCGGAGTGCGCCCTCGGGCGTCGCACCGTGCGGGCTAACGAACCCCGGCGCGGCA   120
var. acuta        TCCCGCCGGAGTGCGCCCTCGGGCGTCGCACCGTGCGGGCTAACGAACCCCGGCGCGGCA   120
var. crispa       TCCCGCCGGAGTGCGCCCTCGGGCGTCGCACCGTGCGGGCTAACGAACCCCGGCGCGGCA   120
var. viridis      TCCCGCCGGAGTGCGCCCTCGGGCGTCGCACCGTGCGGGCTAACGAACCCCGGCGCGGCA   120
                  ************************************************************

var. japonica     AGCGCCAAGGAAAACAAAATTTAGCGCCCGCCTTCCGCATCCCGTTCGCGGGGTGTGCGG   180
var. acuta        AGCGCCAAGGAAAACAAAATTTAGCGCCCGCCTTCCGCATCCCGTTCGCGGGGTGTGCGG   180
var. crispa       AGCGCCAAGGAAAACAAAATTTAGCGCCCGCCTTCCGCATCCCGTTCGCGGGGTGTGCGG   180
var. viridis      AGCGCCAAGGAAAACAAAATTTAGCGCCCGCCTTCCGCATCCCGTTCGCGGGGTGTGCGG   180
                  ************************************************************

var. japonica     GGGGAATGGACGTCTATCGAATGTCATAACGACTCTCGGCAACGGATATCTCGGCTCTCG   240
var. acuta        GGGGAATGGACGTCTATCGAATGTCATAACGACTCTCGGCAACGGATATCTCGGCTCTCG   240
var. crispa       GGGGAATGGACGTCTATCGAATGTCATAACGACTCTCGGCAACGGATATCTCGGCTCTCG   240
var. viridis      GGGGAATGGACGTCTATCGAATGTCATAACGACTCTCGGCAACGGATATCTCGGCTCTCG   240
                  ************************************************************

var. japonica     CATCGATGAAGAACGTAGCGAAATGCGATACTTGGTGTGAATTGCAGAATCCCGTGAACC   300
var. acuta        CATCGATGAAGAACGTAGCGAAATGCGATACTTGGTGTGAATTGCAGAATCCCGTGAACC   300
var. crispa       CATCGATGAAGAACGTAGCGAAATGCGATACTTGGTGTGAATTGCAGAATCCCGTGAACC   300
var. viridis      CATCGATGAAGAACGTAGCGAAATGCGATACTTGGTGTGAATTGCAGAATCCCGTGAACC   300
                  ************************************************************

var. japonica     ATCGAGTCTTTGAACGCAAGTTGCGCCCGAAGCCATTAGGCCGAGGGCACGTCTGCCTGG   360
var. acuta        ATCGAGTCTTTGAACGCAAGTTGCGCCCGAAGCCATTAGGCCGAGGGCACGTCTGCCTGG   360
var. crispa       ATCGAGTCTTTGAACGCAAGTTGCGCCCGAAGCCATTAGGCCGAGGGCACGTCTGCCTGG   360
var. viridis      ATCGAGTCTTTGAACGCAAGTTGCGCCCGAAGCCATTAGGCCGAGGGCACGTCTGCCTGG   360
                  ************************************************************

var. japonica     GCGTCACGCATCGCGTCGCCCCCCTCCCCGCGCTGAGCGCTCGTGAGGGGGGCGGATATT   420
var. acuta        GCGTCACGCATCGCGTCGCCCCCCTCCCCGCGCTGAGCGCTCGTGAGGGGGGCGGATATT   420
var. crispa       GCGTCACGCATCGCGTCGCCCCCCTCCCCGCGCTGAGCGCTCGTGAGGGGGGCGGATATT   420
var. viridis      GCGTCACGCATCGCGTCGCCCCCCTCCCCGCGCTGAGCGCTCGTGAGGGGGGCGGATATT   420
                  ************************************************************

var. japonica     GGCCCCCCGTGCGCCCTGGCGTGCGGTCGGCCCAAATGCGATCCCTCGACGACTCGTGTC   480
var. acuta        GGCCCCCCGTGCGCCCTGGCGTGCGGTCGGCCCAAATGCGATCCCTCGACGACTCGTGTC   480
var. crispa       GGCCCCCCGTGCGCCCTGGCGTGCGGTCGGCCCAAATGCGATCCCTCGACGACTCGTGTC   480
var. viridis      GGCCCCCCGTGCGCCCTGGCGTGCGGTCGGCCCAAATGCGATCCCTCGACGACTCGTGTC   480
                  ************************************************************
```

```
var. japonica      GCGACTAGTGGTGGTTGAATAGCTCAATCTCGTGTCTTGTCGTGCTACCGCGTCGTCCGA   540
var. acuta         GCGACTAGTGGTGGTTGAATAGCTCAATCTCGTGTCTTGTCGTGCTACCGCGTCGTCCGA   540
var. crispa        GCGACTAGTGGTGGTTGAATAGCTCAATCTCGTGTCTTGTCGTGCTACCGCGTCGTCCGA   540
var. viridis       GCGACTAGTGGTGGTTGAATAGCTCAATCTCGTGTCTTGTCGTGCTACCGCGTCGTCCGA   540
                   ************************************************************

var. japonica      ATGGGGATCGAACAACGACCCAACGGTGTTCGTGCGTTACCGCACCGCACCTTCGACCGC   600
var. acuta         ATGGGAATCGAACAACGACCCAACGGTGTTCGTGCGTTACCGCACCGCACCTTCGACCGC   600
var. crispa        ATGGGAATCGAACAACGACCCAACGGTGTTCGTGCGTTACCGCACCGCACCTTCGACCGC   600
var. viridis       ATGGGAATCGAACAACGACCCAACGGTGTTCGTGCGTTACCGCACCGCACCTTCGACCGC   600
                   ***** ******************************************************
```

b)

```
var. japonica      CGATCTATTCATTCAATATTTCCCTTTTTAGAGGACAATTTTTCACATTTAAATTTTGTG  60
var. acuta         CGATCTATTCATTCAATATTTCCCTTTTTAGAGGACAATTTTTCACATTTAAATTTTGTG  60
var. crispa        CGATCTATTCATTCAATATTTCCCTTTTTAGAGGACAATTTTTCACATTTAAATTTTGTG  60
var. viridis       CGATCTATTCATTCAATATTTCCCTTTTTAGAGGACAATTTTTCACATTTAAATTTTGTG  60
                   ************************************************************

var. japonica      TTAGATATACTAATACCTCACTCTGTCCATGCGGAAATCTTGATTCAAACTATTCGCTGT   120
var. acuta         TTAGATATACTAATACCTCACTCTGTCCATGCGGAAATCTTGATTCAAACTATTCGCTGT   120
var. crispa        TTAGATATACTAATACCTCACTCTGTCCATGCGGAAATCTTGATTCAAACTATTCGCTGT   120
var. viridis       TTAGATATACTAATACCTCACTCTGTCCATGCGGAAATCTTGATTCAAACTATTCGCTGT   120
                   ************************************************************

var. japonica      TGGGTAAAAGATGTTTCTTCTTTGCATTTATTACGAGTCTTTCTCAATGAATATTGTAAT   180
var. acuta         TGGGTAAAAGATGTTTCTTCTTTGCATTTATTACGAGTCTTTCTCAATGAATATTGTAAT   180
var. crispa        TGGGTAAAAGATGTTTCTTCTTTGCATTTATTACGAGTCTTTCTCAATGAATATTGTAAT   180
var. viridis       TGGGTAAAAGATGTTTCTTCTTTGCATTTATTACGAGTCTTTCTCAATGAATATTGTAAT   180
                   ************************************************************

var. japonica      TGGAATAGTCTTATTACTCCAAAGAAAGTAAGCTCCTCTTTGTCAAAAAGAAATCAAAGA   240
var. acuta         TGGAATAGTCTTATTACTCCAAAGAAAGTAAGCTCCTCTTTGTCAAAAAGAAATCAAAGA   240
var. crispa        TGGAATAGTCTTATTACTCCAAAGAAAGTAAGCTCCTCTTTGTCAAAAAGAAATCAAAGA   240
var. viridis       TGGAATAGTCTTATTACTCCAAAGAAAGTAAGCTCCTCTTTGTCAAAAAGAAATCAAAGA   240
                   ************************************************************

var. japonica      CTCTTTTTTTTCTTATATAATTCTTACGTATGTGAATACGAATCTGTTTTCGTCTTTCTA   300
var. acuta         CTCTTTTTTTTCTTATATAATTCTTACGTATGTGAATACGAATCTGTTTTCGTCTTTCTA   300
var. crispa        CTCTTTTTTTTCTTATATAATTCTTACGTATGTGAATACGAATCTGTTTTCGTCTTTCTA   300
var. viridis       CTCTTTTTTTTCTTATATAATTCTTACGTATGTGAATACGAATCTGTTTTCGTCTTTCTA   300
                   ************************************************************

var. japonica      CGTAACCAATCTTTTCATTTACGATCAACATCTTCTGGAGTTCTTCTTGAACGAATCTAT   360
var. acuta         CGTAACCAATCTTTTCATTTACGATCAACATCTTCTGGAGTTCTTCTTGAACGAATCTAT   360
var. crispa        CGTAACCAATCTTTTCATTTACGATCAACATCTTCTGGAGTTCTTCTTGAACGAATCTAT   360
var. viridis       CGTAACCAATCTTTTCATTTACGATCAACATCTTCTGGAGTTCTTCTTGAACGAATCTAT   360
                   ************************************************************

var. japonica      TTTTATATAAAAATAAAAACAGAACGTCTTGTGAACGTCTTTGTTTTTGTTAAGGATTTG   420
var. acuta         TTTTATATAAAAATAAAAACAGAACGTCTTGTGAACGTCTTTGTTTTTGTTAAGGATTTG   420
var. crispa        TTTTATATAAAAATAAAAACAGAACGTCTTGTGAACGTCTTTGTTTTTGTTAAGGATTTG   420
var. viridis       TTTTATATAAAAATAAAAACAGAACGTCTTGTGAACGTCTTTGTTTTTGTTAAGGATTTG   420
                   ************************************************************

var. japonica      CGGGCGAACCTAGGGTTGCTCGAGGAACCCTGTATGCATTATATTAGGTATCAAAGAAAA   480
var. acuta         CGGGCGAACCTAGGGTTGCTCGAGGAACCCTGTATGCATTATATTAGGTATCAAAGAAAA   480
var. crispa        CGGGCGAACCTAGGGTTGCTCGAGGAACCCTGTATGCATTATATTAGGTATCAAAGAAAA   480
```

```
var. viridis       CGGGCGAACCTAGGGTTGCTCGAGGAACCCTGTATGCATTATATTAGGTATCAAAGAAAA  480
                   ************************************************************

var. japonica      TCCATTCTGGCTTCAAAAAGGACATCTCTTTTCATGAATAAATGGAAATTTTACCTTGTC  540
var. acuta         TCCATTCTGGCTTCAAAAAGGACATCTCTTTTCATGAATAAATGGAAATTTTACCTTGTC  540
var. crispa        TCCATTCTGGCTTCAAAAAGGACATCTCTTTTCATGAATAAATGGAAATTTTACCTTGTC  540
var. viridis       TCCATTCTGGCTTCAAAAAGGACATCTCTTTTCATGAATAAATGGAAATTTTACCTTGTC  540
                   ************************************************************

var. japonica      ACTTTTTGGCAATGGCATTTTTCGGTGTGGTTTCATCCAAGAAGGATTTGGATAAATCAA  600
var. acuta         ACTTTTTGGCAATGGCATTTTTCGGTGTGGTTTCATCCAAGAAGGATTTGGATAAATCAA  600
var. crispa        ACTTTTTGGCAATGGCATTTTTCGGTGTGGTTTCATCCAAGAAGGATTTGGATAAATCAA  600
var. viridis       ACTTTTTGGCAATGGCATTTTTCGGTGTGGTTTCATCCAAGAAGGATTTGGATAAATCAA  600
                   ************************************************************

var. japonica      TTTTCCAAGCATTCCCTTGAAATTTTGGGCTATCTTTCAAACGTGCAAACGAACCCTTCC  660
var. acuta         TTTTCCAAGCATTCCCTTGAAATTTTGGGCTATCTTTCAAACGTGCAAACGAACCCTTCC  660
var. crispa        TTTTCCAAGCATTCCCTTGAAATTTTGGGCTATCTTTCAAACGTGCAAACGAACCCTTCC  660
var. viridis       TTTTCCAAGCATTCCCTTGAAATTTTGGGCTATCTTTCAAACGTGCAAACGAACCCTTCC  660
                   ************************************************************

var. japonica      GTCGTGGTACGGAGTCAAATTCTAGAAAATTCATTTCTAATCAATAATACTATTAAGAAG  720
var. acuta         GTCGTGGTACGGAGTCAAATTCTAGAAAATTCATTTCTAATCAATAATACTATTAAGAAG  720
var. crispa        GTCGTGGTACGGAGTCAAATTCTAGAAAATTCATTTCTAATCAATAATACTATTAAGAAG  720
var. viridis       GTCGTGGTACGGAGTCAAATTCTAGAAAATTCATTTCTAATCAATAATACTATTAAGAAG  720
                   ************************************************************

var. japonica      CTCGATACCCTTGTTCCAATTATTCCTCTGATTGCGAAATTGGCTAAAGCGAAATTTTGT  780
var. acuta         CTCGATACCCTTGTTCCAATTATTCCTCTGATTGCGAAATTGGCTAAAGCGAAATTTTGT  780
var. crispa        CTCGATACCCTTGTTCCAATTATTCCTCTGATTGCGAAATTGGCTAAAGCGAAATTTTGT  780
var. viridis       CTCGATACCCTTGTTCCAATTATTCCTCTGATTGCGAAATTGGCTAAAGCGAAATTTTGT  780
                   ************************************************************

var. japonica      AATGTATTGGGGCATCCTATTAGTAAGCCGGTTCGGGCTGATTTATCAGATTCTAATATT  840
var. acuta         AATGTATTGGGGCATCCTATTAGTAAGCCGGTTCGGGCTGATTTATCAGATTCTAATATT  840
var. crispa        AATGTATTGGGGCATCCTATTAGTAAGCCGGTTCGGGCTGATTTATCAGATTCTAATATT  840
var. viridis       AATGTATTGGGGCATCCTATTAGTAAGCCGGTTCGGGCTGATTTATCAGATTCTAATATT  840
                   ************************************************************

var. japonica      ATTGACCGATTTGGGCATATATGCAGAAATCTTTCTCATTATTATAGCGGATCTTCCAAA  900
var. acuta         ATTGACCGATTTGGGCATATATGCAGAAATCTTTCTCATTATTATAGCGGATCTTCCAAA  900
var. crispa        ATTGACCGATTTGGGCATATATGCAGAAATCTTTCTCATTATTATAGCGGATCTTCCAAA  900
var. viridis       ATTGACCGATTTGGGCATATATGCAGAAATCTTTCTCATTATTATAGCGGATCTTCCAAA  900
                   ************************************************************

var. japonica      AAAAAGAGTTTGTATCGAATAAATTATATACTTCGACTTTCGTGTGCTAGA 951
var. acuta         AAAAAGAGTTTGTATCGAATAAATTATATACTTCGACTTTCGTGTGCTAGA 951
var. crispa        AAAAAGAGTTTGTATCGAATAAATTATATACTTCGACTTTCGTGTGCTAGA 951
var. viridis       AAAAAGAGTTTGTATCGAATAAATTATATACTTCGACTTTCGTGTGCTAGA 951
                   ***************************************************
```

c)

```
var. japonica      ATGTCACCACAAACAGAAACTAAAGCAAGTGTTGGATTCAAAGCGGGTGTTAAAGAGTAC 60
var. acuta         ATGTCACCACAAACAGAAACTAAAGCAAGTGTTGGATTCAAAGCGGGTGTTAAAGAGTAC 60
var. crispa        ATGTCACCACAAACAGAAACTAAAGCAAGTGTTGGATTCAAAGCGGGTGTTAAAGAGTAC 60
var. viridis       ATGTCACCACAAACAGAAACTAAAGCAAGTGTTGGATTCAAAGCGGGTGTTAAAGAGTAC 60
```

****

var. *japonica* AAATTGACTTATTATACTCCTGAATACGAAACCAAAGATACTGATATCTTGGCAGCATTT 120
var. *acuta* AAATTGACTTATTATACTCCTGAATACGAAACCAAAGATACTGATATCTTGGCAGCATTT 120
var. *crispa* AAATTGACTTATTATACTCCTGAATACGAAACCAAAGATACTGATATCTTGGCAGCATTT 120
var. *viridis* AAATTGACTTATTATACTCCTGAATACGAAACCAAAGATACTGATATCTTGGCAGCATTT 120
****

var. *japonica* CGAGTAACTCCTCAACCTGGAGTTCCGCCTGAAGAAGCAGGGGCCGCGGTAGCTGCCGAA 180
var. *acuta* CGAGTAACTCCTCAACCTGGAGTTCCGCCTGAAGAAGCAGGGGCCGCGGTAGCTGCCGAA 180
var. *crispa* CGAGTAACTCCTCAACCTGGAGTTCCGCCTGAAGAAGCAGGGGCCGCGGTAGCTGCCGAA 180
var. *viridis* CGAGTAACTCCTCAACCTGGAGTTCCGCCTGAAGAAGCAGGGGCCGCGGTAGCTGCCGAA 180
****

var. *japonica* TCTTCTACTGGTACATGGACAACTGTGTGGACCGATGGACTTACCAGCCTTGATCGTTAC 240
var. *acuta* TCTTCTACTGGTACATGGACAACTGTGTGGACCGATGGACTTACCAGCCTTGATCGTTAC 240
var. *crispa* TCTTCTACTGGTACATGGACAACTGTGTGGACCGATGGACTTACCAGCCTTGATCGTTAC 240
var. *viridis* TCTTCTACTGGTACATGGACAACTGTGTGGACCGATGGACTTACCAGCCTTGATCGTTAC 240
****

var. *japonica* AAAGGGCGATGCTACCACATCGAGCCCGTTATTGGAGAAAAAGATCAATATATCTGTTAT 300
var. *acuta* AAAGGGCGATGCTACCACATCGAGCCCGTTATTGGAGAAAAAGATCAATATATCTGTTAT 300
var. *crispa* AAAGGGCGATGCTACCACATCGAGCCCGTTATTGGAGAAAAAGATCAATATATCTGTTAT 300
var. *viridis* AAAGGGCGATGCTACCACATCGAGCCCGTTATTGGAGAAAAAGATCAATATATCTGTTAT 300
****

var. *japonica* GTAGCTTACCCTTTAGACCTTTTTGAAGAAGGTTCTGTTACTAACATGTTTACTTCCATT 360
var. *acuta* GTAGCTTACCCTTTAGACCTTTTTGAAGAAGGTTCTGTTACTAACATGTTTACTTCCATT 360
var. *crispa* GTAGCTTACCCTTTAGACCTTTTTGAAGAAGGTTCTGTTACTAACATGTTTACTTCCATT 360
var. *viridis* GTAGCTTACCCTTTAGACCTTTTTGAAGAAGGTTCTGTTACTAACATGTTTACTTCCATT 360
****

var. *japonica* GTAGGAAATGTATTTGGATTCAAAGCCCTACGTGCTCTACGTCTGGAAGATCTGCGAATT 420
var. *acuta* GTAGGAAATGTATTTGGATTCAAAGCCCTACGTGCTCTACGTCTGGAAGATCTGCGAATT 420
var. *crispa* GTAGGAAATGTATTTGGATTCAAAGCCCTACGTGCTCTACGTCTGGAAGATCTGCGAATT 420
var. *viridis* GTAGGAAATGTATTTGGATTCAAAGCCCTACGTGCTCTACGTCTGGAAGATCTGCGAATT 420
****

var. *japonica* CCTACTGCTTATATTAAAACTTTCCAAGGCCCGCCTCATGGGATCCAAGTTGAGAGAGAT 480
var. *acuta* CCTACTGCTTATATTAAAACTTTCCAAGGCCCGCCTCATGGGATCCAAGTTGAGAGAGAT 480
var. *crispa* CCTACTGCTTATATTAAAACTTTCCAAGGCCCGCCTCATGGGATCCAAGTTGAGAGAGAT 480
var. *viridis* CCTACTGCTTATATTAAAACTTTCCAAGGCCCGCCTCATGGGATCCAAGTTGAGAGAGAT 480
****

var. *japonica* AAATTAAACAAGTACGGTCGTCCTCTGCTGGGATGTACTATTAAACCTAAATTGGGGTTA 540
var. *acuta* AAATTAAACAAGTACGGTCGTCCTCTGCTGGGATGTACTATTAAACCTAAATTGGGGTTA 540
var. *crispa* AAATTAAACAAGTACGGTCGTCCTCTGCTGGGATGTACTATTAAACCTAAATTGGGGTTA 540
var. *viridis* AAATTAAACAAGTACGGTCGTCCTCTGCTGGGATGTACTATTAAACCTAAATTGGGGTTA 540
****

var. *japonica* TCTGCTAAAAACTATGGTAGAGCGGTTTATGAATGTCTTCGCGGTGGACTTGATTTTACC 600
var. *acuta* TCTGCTAAAAACTATGGTAGAGCGGTTTATGAATGTCTTCGCGGTGGACTTGATTTTACC 600
var. *crispa* TCTGCTAAAAACTATGGTAGAGCGGTTTATGAATGTCTTCGCGGTGGACTTGATTTTACC 600
var. *viridis* TCTGCTAAAAACTATGGTAGAGCGGTTTATGAATGTCTTCGCGGTGGACTTGATTTTACC 600
****

var. *japonica* AAAGATGATGAGAACGTGAACTCCCAGCCATTTATGCGTTGGAGAGACCGCTTCTTATTT 660
var. *acuta* AAAGATGATGAGAACGTGAACTCCCAGCCATTTATGCGTTGGAGAGACCGCTTCTTATTT 660

```
var. crispa      AAAGATGATGAGAACGTGAACTCCCAGCCATTTATGCGTTGGAGAGACCGCTTCTTATTT   660
var. viridis     AAAGATGATGAGAACGTGAACTCCCAGCCATTTATGCGTTGGAGAGACCGCTTCTTATTT   660
                 ************************************************************

var. japonica    TGTGCCGAAGCAATTTATAAAGCACAGGCTGAAACAGGTGAAATTAAAGGGCATTACTTG   720
var. acuta       TGTGCCGAAGCAATTTATAAAGCACAGGCTGAAACAGGTGAAATTAAAGGGCATTACTTG   720
var. crispa      TGTGCCGAAGCAATTTATAAAGCACAGGCTGAAACAGGTGAAATTAAAGGGCATTACTTG   720
var. viridis     TGTGCCGAAGCAATTTATAAAGCACAGGCTGAAACAGGTGAAATTAAAGGGCATTACTTG   720
                 ************************************************************

var. japonica    AATGCTACTGCAGGTACATGCGA         743
var. acuta       AATGCTACTGCAGGTACATGCGA         743
var. crispa      AATGCTACTGCAGGTACATGCGA         743
var. viridis     AATGCTACTGCAGGTACATGCGA         743
                 ***********************
```

d)

```
var. japonica    GTTATGCATGAACGTAATGCTCATAATTTCCCTCTAGACCTAGCTGCTATCGAAGCTCCA  60
var. acuta       GTTATGCATGAACGTAATGCTCATAATTTCCCTCTAGACCTAGCTGCTATCGAAGCTCCA  60
var. crispa      GTTATGCATGAACGTAATGCTCATAATTTCCCTCTAGACCTAGCTGCTATCGAAGCTCCA  60
var. viridis     GTTATGCATGAACGTAATGCTCATAATTTCCCTCTAGACCTAGCTGCTATCGAAGCTCCA  60
                 ************************************************************

var. japonica    ACAAATGGCTAAGACTTGTTCTTAGTGTGTAGGAGTTTTTGAAAATAGAATAGATAAATA   120
var. acuta       ACAAATGGCTAAGACTTGTTCTTAGTGTGTAGGAGTTTTTGAAAATAGAATAGATAAATA   120
var. crispa      ACAAATGGCTAAGACTTGTTCTTAGTGTGTAGGAGTTTTTGAAAATAGAATAGATAAATA   120
var. viridis     ACAAATGGCTAAGACTTGTTCTTAGTGTGTAGGAGTTTTTGAAAATAGAATAGATAAATA   120
                 ************************************************************

var. japonica    TAAGGAGCAATAAACCCCTTCTTGTTCTATCAAAAGAGGGTTTATTGCTCCTTCATTTTC   180
var. acuta       TAAGGAGCAATAAACCCCTTCTTGTTCTATCAAAAGAGGGTTTATTGCTCCTTCATTTTC   180
var. crispa      TAAGGAGCAATAAACCCCTTCTTGTTCTATCAAAAGAGGGTTTATTGCTCCTTCATTTTC   180
var. viridis     TAAGGAGCAATAAACCCCTTCTTGTTCTATCAAAAGAGGGTTTATTGCTCCTTCATTTTC   180
                 ************************************************************

var. japonica    TTTTCAATTAGGAATCTTTTTCTATTTTTTCTAGTAGTATTGGACTTATCTAGATTTTTT   240
var. acuta       TTTTCAATTAGGAATCTTTTTCTATTTTTTCTAGTAGTATTGGACTTATCTAGATTTTTT   240
var. crispa      TTTTCAATTAGGAATCTTTTTCTATTTTTTCTAGTAGTATTGGACTTATCTAGATTTTTT   240
var. viridis     TTTTCAATTAGGAATCTTTTTCTATTTTTTCTAGTAGTATTGGACTTATCTAGATTTTTT   240
                 ************************************************************

var. japonica    CTTTCCATTATAGAAAAAGAAAGAAGATAAAAATGATTCAAATTCCATGTTTTGTTTTAC   300
var. acuta       CTTTCCATTATAGAAAAAGAAAGAAGATAAAAATGATTCAAATTCCATGTTTTGTTTTAC   300
var. crispa      CTTTCCATTATAGAAAAAGAAAGAAGATAAAAATGATTCAAATTCCATGTTTTGTTTTAC   300
var. viridis     CTTTCCATTATAGAAAAAGAAAGAAGATAAAAATGATTCAAATTCCATGTTTTGTTTTAC   300
                 ************************************************************

var. japonica    AATTTCTAAAAAAATTCAAATTGAAAAAGTGAATTCGTAAATAAAAAGTCAATCAATTTT   360
var. acuta       AATTTCTAAAAAAATTCAAATTGAAAAAGTGAATTCGTAAATAAAAAGTCAATCAATTTT   360
var. crispa      AATTTCTAAAAAAATTCAAATTGAAAAAGTGAATTCGTAAATAAAAAGTCAATCAATTTT   360
var. viridis     AATTTCTAAAAAAATTCAAATTGAAAAAGTGAATTCGTAAATAAAAAGTCAATCAATTTT   360
                 ************************************************************

var. japonica    GAATTTAATATAAATTTTTCTAATATTAACTCTAAATCTTTCTATAGTAGAGGGGCGGAT   420
var. acuta       GAATTTAATATAAATTTTTCTAATATTAACTCTAAATCTTTCTATAGTAGAGGGGCGGAT   420
```

```
var. crispa      GAATTTAATATAAATTTTTCTAATATTAACTCTAAATCTTTCTATAGTAGAGGGGCGGAT   420
var. viridis     GAATTTAATATAAATTTTTCTAATATTAACTCTAAATCTTTCTATAGTAGAGGGGCGGAT   420
                 ************************************************************

var. japonica    GTAGCCAAGTGGATCAAGGCAGTGGATTGTGAATCCACCATGCGCG       466
var. acuta       GTAGCCAAGTGGATCAAGGCAGTGGATTGTGAATCCACCATGCGCG       466
var. crispa      GTAGCCAAGTGGATCAAGGCAGTGGATTGTGAATCCACCATGCGCG       466
var. viridis     GTAGCCAAGTGGATCAAGGCAGTGGATTGTGAATCCACCATGCGCG       466
                 **********************************************
```

e)

```
var. japonica    TCGATTATTCAGGGCGTTCCGTCATTGTCGTAGGTCCTTCACTTTCATTACACCGGTGTG  60
var. crispa      TCGATTATTCAGGGCGTTCCGTCATTGTCGTAGGTCCTTCACTTTCATTACACCGGTGTG  60
var. acuta       TCGATTATTCAGGGCGTTCCGTCATTGTCGTAGGTCCTTCACTTTCATTACACCGGTGTG  60
var. viridis     TCGATTATTCAGGGCGTTCCGTCATTGTCGTAGGTCCTTCACTTTCATTACACCGGTGTG  60
                 ************************************************************

var. japonica    GATTGCCGCGGGAAATAGCAATAGAACTTTTCCAGACGTTTGTAATTCGTGGTCTAATTA   120
var. crispa      GATTGCCGCGGGAAATAGCAATAGAACTTTTCCAGACGTTTGTAATTCGTGGTCTAATTA   120
var. acuta       GATTGCCGCGGGAAATAGCAATAGAACTTTTCCAGACGTTTGTAATTCGTGGTCTAATTA   120
var. viridis     GATTGCCGCGGGAAATAGCAATAGAACTTTTCCAGACGTTTGTAATTCGTGGTCTAATTA   120
                 ************************************************************

var. japonica    GACAACATCTTGCTTCGAACATAGGAGTTGCTAAGAGTAAAATTCGGGGAAAAGAACTGA   180
var. crispa      GACAACATCTTGCTTCGAACATAGGAGTTGCTAAGAGTAAAATTCGGGGAAAAGAACTGA   180
var. acuta       GACAACATCTTGCTTCGAACATAGGAGTTGCTAAGAGTAAAATTCGGGGAAAAGAACTGA   180
var. viridis     GACAACATCTTGCTTCGAACATAGGAGTTGCTAAGAGTAAAATTCGGGGAAAAGAACTGA   180
                 ************************************************************

var. japonica    TTGTATGGGAAATATTGCAGGAAGTTATGCAGGGGCATCCTGTATTGCTGAATAGAGCCC   240
var. crispa      TTGTATGGGAAATATTGCAGGAAGTTATGCAGGGGCATCCTGTATTGCTGAATAGAGCCC   240
var. acuta       TTGTATGGGAAATATTGCAGGAAGTTATGCAGGGGCATCCTGTATTGCTGAATAGAGCCC   240
var. viridis     TTGTATGGGAAATATTGCAGGAAGTTATGCAGGGGCATCCTGTATTGCTGAATAGAGCCC   240
                 ************************************************************

var. japonica    CCACTCTGCATAAATTGGGCATACAGGCATTCCAGCCCGTTTTAGTGGAGGGGCGTGCTA   300
var. crispa      CCACTCTGCATAAATTGGGCATACAGGCATTCCAGCCCGTTTTAGTGGAGGGGCGTGCTA   300
var. acuta       CCACTCTGCATAAATTGGGCATACAGGCATTCCAGCCCGTTTTAGTGGAGGGGCGTGCTA   300
var. viridis     CCACTCTGCATAAATTGGGCATACAGGCATTCCAGCCCGTTTTAGTGGAGGGGCGTGCTA   300
                 ************************************************************

var. japonica    TTTGTTTACATCCATTAGTTTGTAAGGGATTCAATGCAGATTTTGATGGGGATCAAATGG   360
var. crispa      TTTGTTTACATCCATTAGTTTGTAAGGGATTCAATGCAGATTTTGATGGGGATCAAATGG   360
var. acuta       TTTGTTTACATCCATTAGTTTGTAAGGGATTCAATGCAGATTTTGATGGGGATCAAATGG   360
var. viridis     TTTGTTTACATCCATTAGTTTGTAAGGGATTCAATGCAGATTTTGATGGGGATCAAATGG   360
                 ************************************************************

var. japonica    CTGTTCATGTACCCTTATCGTTGGAGGCTCAAGCGGAGGCCCGTTTACTTATGTTTTCTC   420
var. crispa      CTGTTCATGTACCCTTATCGTTGGAGGCTCAAGCGGAGGCCCGTTTACTTATGTTTTCTC   420
var. acuta       CTGTTCATGTACCCTTATCGTTGGAGGCTCAAGCGGAGGCCCGTTTACTTATGTTTTCTC   420
var. viridis     CTGTTCATGTACCCTTATCGTTGGAGGCTCAAGCGGAGGCCCGTTTACTTATGTTTTCTC   420
                 ************************************************************

var. japonica    ATATGAATCTTTTGTCTCCAGCTATTGGAGATCCCATTTCCGTACCAACTCAAGATATGC   480
var. crispa      ATATGAATCTTTTGTCTCCAGCTATTGGAGATCCCATTTCCGTACCAACTCAAGATATGC   480
var. acuta       ATATGAATCTTTTGTCTCCAGCTATTGGAGATCCCATTTCCGTACCAACTCAAGATATGC   480
var, viridis     ATATGAATCTTTTGTCTCCAGCTATTGGAGATCCCATTTCCGTACCAACTCAAGATATGC   480
```

```
                    ************************************************************

var. japonica       TTATGAACCCCA         492
var. crispa         TTATGAAAAAAG         492
var. acuta          TTATGGAAAACA         492
var. viridis        TTATGGAACAA-         491
                    ***** *
```

f)

```
var. viridis        TTTTCACTGTTTCATTCTATTACAATTCATTTGATAACCTTTTTTGTCGATGAAATCATA  60
var. crispa         TTTTCGCTGTTTCATTCTATTACAATTCATTTGATAACCTTTTTTGTCGATGAAATCATA  60
var. japonica       TTTTCGCTGTT-TATTCTATTACAATTCATTTGATAACCTTTTTTGTCGATGAAATCATA  59
var. acuta          TTTTCGCTGTTTCATTCTATTACAATTCATTTGATAACCTTTTTTGTCGATGAAATCATA  60
                    ***** ***** ************************************************

var. viridis        AGATTATATGATTCATCCGAAAGGGGCATGATAATCACCTTTTTGTGTATAACAGGATTA  120
var. crispa         AGATTATATGATTCATCCGAAAGGGGCATGATAATCACCTTTTTGTGTATAACAGGATTA  120
var. japonica       AGATTATATGATTCATCCGAAAGGGGCATGATAATCACCTTTTTGTGTATAACAGGATTA  119
var. acuta          AGATTATATGATTCATCCGAAAGGGGCATGATAATCACCTTTTTGTGTATAACAGGATTA  120
                    ************************************************************

var. viridis        TTAATTTCTCGTTGGATTTATTCAAAACATTTTCCACTAAGTGATTTATATGAATCGTTA  180
var. crispa         TTAATTTCTCGTTGGATTTATTCAAAACATTTTCCACTAAGTGATTTATATGAATCGTTA  180
var. japonica       TTAATTTCTCGTTGGATTTATTCAAAACATTTTCCACTAAGTGATTTATATGAATCGTTA  179
var. acuta          TTAATTTCTCGTTGGATTTATTCAAAACATTTTCCACTAAGTGATTTATATGAATCGTTA  180
                    ************************************************************

var. viridis        ATCTTTCTTTCATGGAGTTTTTCCTTTATTTATATAGTTCCGTATTTCAAAAAAAATCAA  240
var. crispa         ATCTTTCTTTCATGGAGTTTTTCCTTTATTTATATAGTTCCGTATTTCAAAAAAAATCAA  240
var. japonica       ATCTTTCTTTCATGGAGTTTTTCCTTTATTTATATAGTTCCGTATTTCAAAAAAAATCAA  239
var. acuta          ATCTTTCTTTCATGGAGTTTTTCCTTTATTTATATAGTTCCGTATTTCAAAAAAAATCAA  240
                    ************************************************************

var. viridis        AATTTTCTAAGCACAATAATAGGTCCAAGTGCTATTTTTTTCCCAGGGTTTTGCTACCTCG  300
var. crispa         AATTTTCTAAGCACAATAATAGGTCCAAGTGCTATTTTTTTCCCAGGGTTTTGCTACCTCG  300
var. japonica       AATTTTCTAAGCACAATAATAGGTCCAAGTGCTATTTTTTTCCCAGGGTTTTGCTACCTCG  299
var. acuta          AATTTTCTAAGCACAATAATAGGTCCAAGTGCTATTTTTTTCCCAGGGTTTTGCTACCTCG  300
                    ************************************************************

var. viridis        GGTCTTTTAACTGAAATACACGAATCCACTATATTAGTACCCGCTCTTCAATCCGAGTGG  360
var. crispa         GGTCTTTTAACTGAAATACACGAATCCACTATATTAGTACCCGCTCTTCAATCCGAGTGG  360
var. japonica       GGTCTTTTAACTGAAATACACGAATCCACTATATTAGTACCCGCTCTTCAATCCGAGTGG  359
var. acuta          GGTCTTTTAACTGAAATACACGAATCCACTATATTAGTACCCGCTCTTCAATCCGAGTGG  360
                    ************************************************************

var. viridis        TTAATGATGCACGTAAGTTTAA          382
var. crispa         TTAATGATGCACGTAAGTTAAA          382
var. japonica       TTAATGATGCACGTAAGTAAAA          381
var. acuta          TTAATGATGCACGTAAGTAAAA          382
                    *****************  **
```
